# An inexpensive, high-throughput μPAD assay of microbial growth rate and motility on solid surfaces using *Saccharomyces cerevisiae* and *Escherichia coli* as model organisms

**DOI:** 10.1371/journal.pone.0225020

**Published:** 2020-10-08

**Authors:** Alyssa Francesca Levy, Anthony Labrador, Leslie Knecht, J. David Van Dyken

**Affiliations:** 1 University of Miami, Coral Gables, FL, United States of America; 2 Department of Chemistry, University of Miami, Coral Gables, FL, United States of America; 3 Department of Biology, University of Miami, Coral Gables, FL, United States of America; University of Illinois at Chicago, UNITED STATES

## Abstract

Many microbial phenotypes are differentially or exclusively expressed on agar surfaces, including biofilms, motility, and sociality. However, agar-based assays are limited by their low throughput, which increases costs, lab waste, space requirements, and the time required to conduct experiments. Here, we demonstrate the use of wax-printed microfluidic paper-based analytical devices (μPADs) to measure linear growth rate of microbes on an agar growth media as a means of circumventing the aforementioned limitations. The main production materials of the proposed μPAD design are a wax printer, filter paper, and empty pipet boxes. A single wax-printed μPAD allowing 8 independent, agar-grown colonies costs $0.07, compared to $0.20 and $9.37 for the same number of replicates on traditional microtiter/spectrophotometry and Petri dish assays, respectively. We optimized the μPAD design for channel width (3 mm), agar volume (780 μL/channel), and microbe inoculation method (razor-blade). Comparative analyses of the traditional and proposed μPAD methods for measuring growth rate of nonmotile (*Saccharomyces cerevisiae*) and motile (flagellated *Escherichia coli*) microorganisms suggested the μPAD assays conferred a comparable degree of accuracy and reliability to growth rate measurements as their traditional counterparts. We substantiated this claim with strong, positive correlations between the traditional and μPAD assay, a significant nonzero slope in the model relating the two assays, a nonsignificant difference between the relative standard errors of the two techniques, and an analysis of inter-device reliability. Therefore, μPAD designs merit consideration for the development of enhanced-throughput, low-cost microbial growth and motility assays.

## Introduction

One of the most widely used assays for microbiological studies is the growth assay in which strains are grown in different environments for gross interrogation of cellular processes. Traditional assays are usually prepared via inoculation on agar media in Petri dishes or suspension in media broth in test tubes or multi-well plates. Liquid culture assays are often preferred over agar-based assays because of their high-throughput via automated plate readers which can be programmed to take periodic optical density (OD) measurements of cultures in 96- or 384-well formats over the entire growth cycle in order to produce a growth curve. However, a major biological limitation of liquid-based assays is that many microbes of interest live attached to surfaces rather than suspended in liquid culture [1, 2]. Consequently, numerous phenotypes of interest are either absent in liquid culture or subject to growth conditions that greatly alter their phenotypic effects [3, 4]. Further, it has been shown that artificial selection of microbes during lab domestication to a regime of serial liquid culture propagation has led to the loss of many ecologically significant traits, particularly those associated with sociality, biofilm formation and motility [5, 6], highlighting how cryptic phenotypic variation may be revealed only when grown on solid agar surfaces. For example, most prokaryotes and yeasts feed by secreting exoenzymes that digest extracellular resources into products that can be acquired by cells via diffusion or transport. It has been demonstrated that growth relying on exoenzyme production is qualitatively different in shaken liquid cultures (such as those in a plate reader) than on solid surfaces [7, 8]. Furthermore, the dynamics of growth on surfaces differs from that in shaken liquid culture due to the emergence of spatial heterogeneity in nutrient availability and cell density within a spatially expanding colony on a surface. Whereas cells in shaken liquid culture experience a homogenous environment, cells grown on a surface experience high crowding with cell-cell contacts that may be important for the expression of biologically relevant traits such as quorum sensing, conjugation, or exopolysaccharide. Cells at the interior of colonies experience much higher cell-cell contact and nutrient stress than cells at the expanding colony frontier, generating heterogeneity in growth rates and gene expression among cells that may be crucial for understanding the behavior and phenotypic diversity of microbial consortia in natural settings [9]. Finally, motility assays are typically performed in agar plates, in part because many motility phenotypes, such as twitching and social swarming motility, are not expressed in liquid culture, and also because motility is easier to measure in macroscopic colonies than at the single-cell level. Traditionally-used agar plates are expensive, bulky, contribute to lab waste, and generally lead to low-throughput assays.

The shortcomings of these preparation and analysis methods for growth rate assays include their high cost and, particularly in the case of motility assays, low-throughput. This proves especially disadvantageous where funding is limited, including labs of developing regions, or in educational settings such as high-school or undergraduate labs. In such circumstances, a microfluidic paper-based device could facilitate the assembly and analysis of inexpensive, high-throughput assays of microbial growth rate and motility. The last decade has seen an influx in research for the development of microfluidic devices. Nie and colleagues [10] developed a microfluidic paper-based electrochemical device (μPED) with paper, tape, electrodes to quantify the concentration of different analytes, including glucose and heavy metals, in aqueous solutions. Similarly, Ellerbee and colleagues [11] integrated a microfluidic paper-based analytical device (μPAD) with a hand-held transmittance calorimeter to detect abiotic analyte concentrations as a function of light transmittance through the μPAD. In fact, several paper-based devices have been engineered for electrochemical assays, such as bacteria-powered batteries [12] and glucose detection via amperometric sensors [13], and the evolution in design, construction, and use of electrochemical paper-based analytical devices is documented in several reviews [14, 15]. Paper-based assays are largely implemented for detection and culture assays, which may serve to indicate the prevalence of a protein or particular microbes in culture. For example, Jokerst and colleagues [16] optimized a wax-printed μPAD assay protocol to determine the prevalence of foodborne pathogens via bacteria-specific enzyme, live bacteria, and food sample analyses in 2012. In the same year, Funes-Huacca and colleagues [17] developed a paper-based platform for a bacterial growth assay in liquid culture. In 2017, Boehle and colleagues [18] demonstrated the use of a wax-printed paper-based assay in the detection of antimicrobial resistance in different bacterial species. For an overview of the modern approaches to paper-based analytical devices, we recommend several literature reviews [19, 20]. It is important to note that, although there are several studies demonstrating microfluidic approaches to monitoring bacterial growth, there are very few papers that describe paper-based platforms to quantify yeast and bacterial growth rate.

Although each of these developments deserve merit for suggesting low-cost methodology for analyzing solutions and possibly pathogenic samples, not much attention has been paid to the possibilities of μPADs for increasing the throughput and decreasing the cost of agar-based microbial growth rate and motility assays. As mentioned, the dynamics of microbial growth on solid surfaces may be markedly different than in liquid media. Thus, our μPAD design addresses this unique concern, specifically with under-developed paper-based yeast growth assays. Most notably, the design embraces an environmentally friendly, cost-efficient, and high-throughput preparation. The main components of the μPAD are recycled pipet boxes and wax-printed filter paper. Carrilho and colleagues [21] estimated the cost of wax-printing 8.5” x 11” Whatman #1 filter paper was $0.001/cm^2. Therefore, the cost of printing a quadruplet set of μPAD channels (4 mm x 11 mm channel interior, pre-melted) for each trial is about $0.0365/cm^2. A standard 8.5 cm x 12 cm x 6 cm pipet-tip box holds two quadruplets, or two trials. This equates to eight petri dishes ($9.47) or eight wells in a non-reusable microtiter plate ($0.20) for traditional growth assays. We determined these prices for those provided for a 60 mm x 15 mm petri dish and 96-well 350 μL plates from Thermo Fisher Scientific.

Of course, both methodologies have much more substantial start-up prices due to the equipment needed. Spectrophotometers, like the Tecan Nanoquant Infinite M200 Pro spectrophotometer we used for assessment of microbial growth in liquid culture, cost between $5000 (pre-owned from eBay Inc.) and $9000 (new from Tecan Trading AG). On the other hand, the wax printer has a much lower start-up price. The Xerox ColorQube 8580 model used to print the wax channels has since been discontinued. A used version of the printer can be purchased for about $500 (PC and More). Of course, alternative styles of wax printing and screening exist [22, 23]. The Xerox ColorQube 8580 printer, as well as the comparable Xerox Phaser 8560N used in other methodologies, doubles as a functional office printer, further substantiating the equipment investment [21]. The ink cartridges for the Xerox ColorQube 8580 printer cost $123 for the black toner cartridge, which yields 4300 pages, and $720 for the color bundle toner cartridges, which yields 4400 to 8600 pages (Xerox Corporation). Regardless of whether equipment is purchased new or used, the start-up cost of the proposed wax-printing methodology is much more economical than its spectrophotometry counterpart.

Additionally, the assay was designed to have a simple, user-friendly analysis method. Microbial growth analysis could be done with the naked eye, a smart phone, or computer digital editing software, depending on the accuracy needed for analysis. Herein, we implemented basic Adobe Photoshop techniques to determine growth rate on the μPAD and petri dish assays. Most importantly, the proposed μPAD method for determining linear growth rate is facile, which allows for untrained individuals to readily prepare the μPAD design and acquire successful results. Further, any researcher can easily modify the platform design to fit his/her analytical needs because we created this platform on the common computer program Microsoft Office Word. Therefore, the combination of low cost, high throughput, and simplicity suggests μPAD assays could be implemented for growth assays with motile and nonmotile organisms.

Here, we investigate the optimization of a protocol for conducting microbial growth rate and motility assays on μPADs. Wax-printed μPADs take advantage of the wax’s hydrophobic properties to direct the spatial distribution of growth medium, and thus microorganisms, in a desired shape and direction. We outline a three-phase process in the development of these μPADs for growth assays. First, we optimized the μPAD parameters and inoculation procedure to maximize growth displacement, while minimizing measurement error. We compared the precision and accuracy of growth rate measurements against those attained by traditional techniques. Second, we manipulated growth rate in *S*. *cerevisiae*, which is nonmotile, by varying the concentration of the growth-inhibiting drug cycloheximide [24]. The linear rate of colony expansion is proportional to the growth rate in *S*. *cerevisiae* [25]. Third, we similarly manipulated motility in *E*. *coli* by varying sucrose concentration [26]. Both yeast and bacteria manipulations allow for analysis of our μPAD method’s sensitivity.

## Methods and materials

### μPAD preparation and optimization

**Fig 1:**
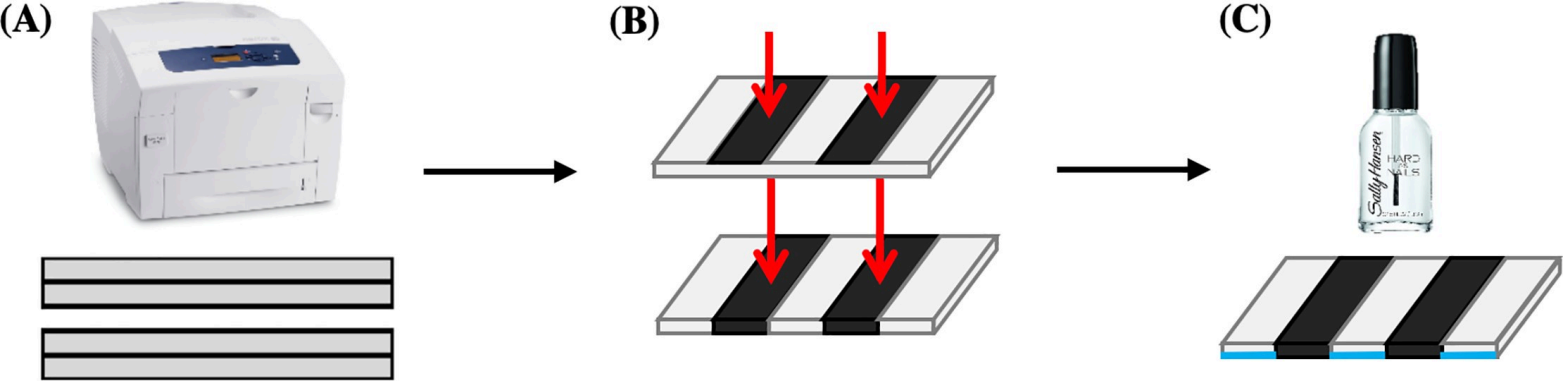
μPAD paper preparation. The schematic represents the preparation of the μPAD. (A) We printed the channels with the Xerox ColorQube 8580. We printed the channels with a width of .4 cm, length of 10.1 cm, and barrier thickness of 3 mm. These dimension account for the wax diffusion that occurs during the melting phase. (B) We melted the channels in an oven set to 100°C. (C) Then, we brushed clear nail polish, denoted by the blue regions, in the unprinted, hydrophilic channels on one side of the paper.

**Fig 2:**
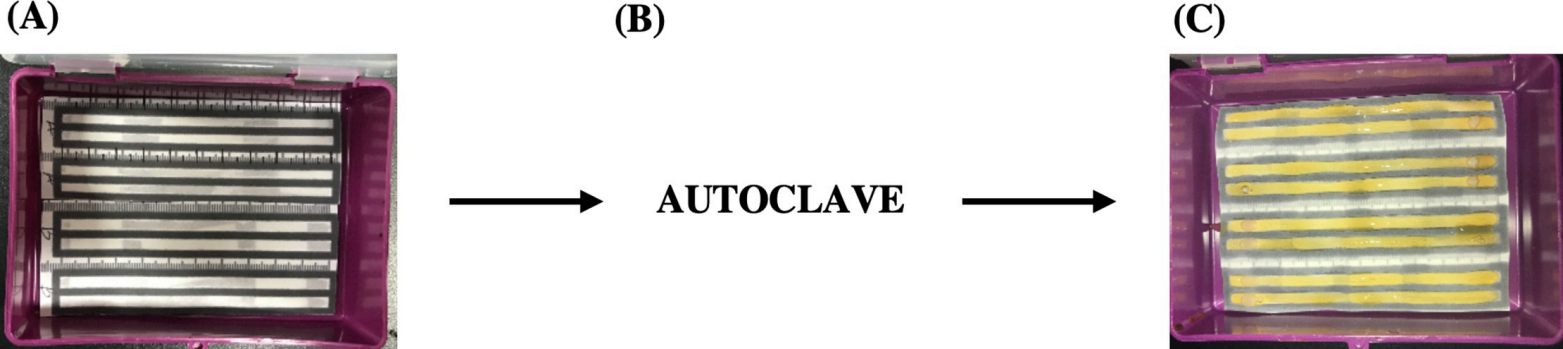
μPAD device construction. The photographs show the preparation of the complete μPAD device. (A) We taped the paper prepared via the methodology presented in Fig 1 to an interior face of an empty, clean pipet box. (B) We autoclaved the closed device and hereafter handled it with sterile technique. (C) We inoculated each channel with agar growth media and cells. After this, we closed the box, inverted the box, and added 50 mL water to the interior region of the bottom portion of the box as a humidity chamber.

We created the channels with Microsoft Word with the “Tables” feature and the specifications for length and width determined by “Row” and “Column” dimensions. We printed the channels onto Whatman #1 filter paper with a Xerox ColorQube 8580 wax printer, and we melted the wax ink in an oven at 100°C for a minimum of 15 min with additional 5 min intervals of heat until the channels melted homogenously thoroughly to the paper and were clearly visible on both sides of each sheet. We coated the hydrophilic (unprinted) areas within each channel with clear nail polish, such as Sally Hanson Double Duty Clear Coat, on one side of the melted sheets to provide a hydrophobic barrier for later media inoculations (Fig 1). The hydrophobicity of nail polish for paper-based analytical devices has been established [27]. We taped the nail-polished side of prepared μPAD sheets into empty, clean pipet-tip boxes with labelling tape and closed the boxes for autoclaving (Fig 2). Hereafter, we handled the boxes with traditional sterile techniques, including a clean bench, open ethanol flame, and gloves; we only opened the boxes to inoculate the agar and cells and to take the appropriate pictures.

Our goal was to maximize the number of replicate assays per 8.5 cm x 12 cm μPAD, which are the dimensions of one face of the pipet-tip boxes used. Therefore, the approach we took was to design linear channels for agar arrayed on a paper surface. We optimized the protocol for developing a μPAD system for channel width, agar volume, and decontamination. Thus, we implemented a 2x3 factorial design of the melted channel width (1.0, 3.0, 5.0 mm) and agar volume parameters to determine the most efficient parameter combination with the lowest error in measurement. We varied agar volumes according to the ratio between channel area and agar volume (μL:mm2), which were tested at 2:1, 1.5:1, and 0.3:1; we predetermined these ratios during a preliminary test at inoculating agar. To prevent as much contamination as possible, we opened the pipet tip boxes in the presence of a flame just enough to inoculate the agar.

**Fig 3:**
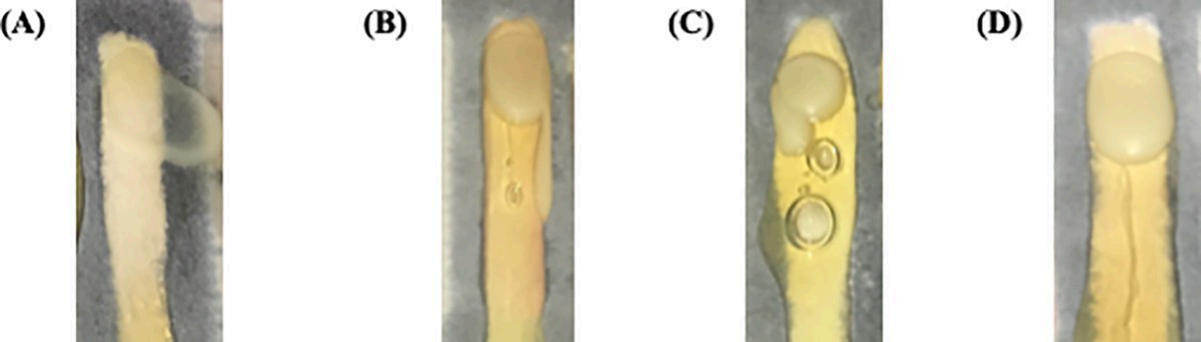
μPAD growth score guide. The Growth Scores quantified the characteristic growth fronts within channels that would influence final growth displacement measurements. (A) Score 0 indicates agar or inoculum spillage from the hydrophobic barriers, rendering data points of one or more channels useless. (B) Score 1 indicates “side growth” where only < 25% of the colony front would grow farther than the rest of the colony along the hydrophobic barrier, making it difficult to determine a point of reference for growth. (C) Score 2 indicates “irregular growth fronts” where an obstruction (air bubble) or accidental inoculation error resulted in a heterogeneous front that does not completely span the width of the channel, making it difficult to determine a point of reference for growth. (D) Score 3 indicates “flat fronts” where the colony would completely span the width of the channel, and the colony front was perpendicular to the hydrophobic barrier. This growth pattern is ideal.

We inoculated the yeast onto the channels (OD660 = 2.0) on Day 0. We tried two microbial inoculation methods, including pipet inoculation (10 μL) and ethanol/flame-sterilized razor blade inoculation. For both methods, we incubated the boxes at 30°C, and recorded growth on Day 1 and Day 5 with a smartphone camera. Again, we minimized contamination by opening the boxes near an open ethanol flame for a limited amount of time (< 2 sec) to take the pictures with an ethanol-sterilized smartphone camera. We determined growth displacement with Adobe Photoshop software. We compared the microbial inoculation methods by scoring the colonial growth fronts on each of the channels (Fig 3). The growth scores were compiled with the growth displacement to determine the most effective combination of channel width and agar growth to be applied in agar-based growth assays on μPAD systems.

### Final assay development

**Fig 4:**
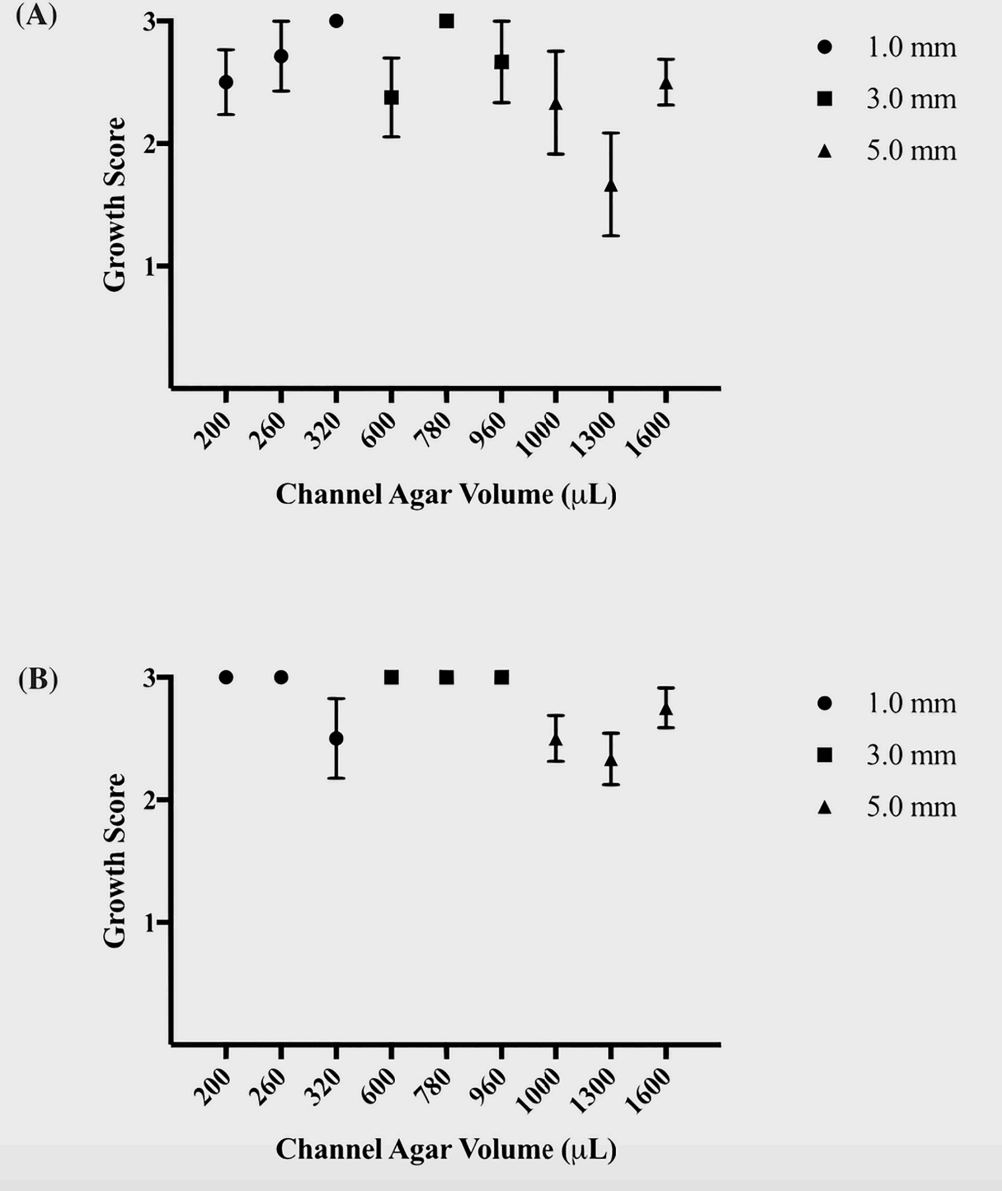
Growth scores for pipet and razor-blade microbial inoculation methods. The tables show the mean growth score with *SEM* bars for microbes inoculated via (A) pipet-inoculation and (B) razor blade-inoculation across different agar volumes. Note the increased number of channels that exhibited a uniform, flat growth front (Score 3) when inoculated via the latter method.

As discussed in the results, the ideal dimensions for the channels as 100 mm x 3 mm with 780 μL of agar growth media. We used these parameters for the final assay development of the *S*. *cerevisiae* and *E*. *coli* sensitivity experiments via the aforementioned preparation protocol (Figs 1 and 2). We washed the *S*. *cerevisiae* and *E*. *coli* three times and then suspended the respective pellets with 3 mL autoclaved DI H2O. We checked the optical density at 660 nm wavelength absorption with the Tecan Nanoquant Infinite M200 Pro spectrophotometer and resuspended the samples with the appropriate amount of DH2O to have an OD660 = 2.0. We plated the appropriate inoculum at one end of the channels, using an ethanol-sterilized razor blade to ensure a flat, uniform growth front (Fig 4). We kept the μPAD platforms with the agar and microbial inoculation in the closed boxes for ~1 hr, or until the inoculum dried, at which point we flipped the boxes so the channels would be on the interior roof of the boxes. We created a humidity chamber by opening the boxes near an open flame just enough to add 50 mL of autoclaved DI H2O to the interior bottoms of the boxes. We sealed the boxes with Parafilm and incubated them at 30°C and 37°C for *S*. *cerevisiae* and *E*. *coli*, respectively.

We recorded colonial growth via the same phone-sterilized protocol mentioned in μPAD Preparation and Optimization. For *S*. *cerevisiae*, we inoculated the microbes on Day 0 and took pictures on Days 1 and 5. For *E*. *coli*, we inoculated the microbes at Hour 0 and took pictures at Hours 6 and 30. We account the differential recording periods to the reduced doubling time of *E*. *coli* compared to *S*. *cerevisiae*. We kept the samples in their respective incubation temperatures between picture documentation. We used Adobe Photoshop software to determine the displacement. Figs 1 and 2 summarize the μPAD and box preparation methods.

### *S*. *cerevisiae* cycloheximide concentration gradient

We made the growth media increasingly toxic to the yeast cells by varying cycloheximide concentrations to determine the μPAD assay’s sensitivity to displacement and growth rate measurement of a non-motile organism. We prepared 100 mL YPD agar (20 g/L Bactopeptone, 10 g/L yeast extract, 10 g/L agar, and 10 g/L dextrose in DH2O; .1% tetracyclin and .1% ampicillin by volume) and segregated the sample into six parts to prepare the various cycloheximide concentrations (0, 200, 400, 600, 800, 1000 nM) for the μPAD assay. We compared the μPAD assay against a “traditional” growth plate assay during which we suspended yeast in a 96-well plate with the appropriate concentrations of cycloheximide in non-agarose YPD. We determined the growth rate of yeast measured in the traditional assay via a growth curve acquired by Tecan Nanoquant Infinite M200 Pro spectrophotometer readings at an optical density of 660 nm (OD660). We determined the growth rate of yeast measured in the μPAD assay by finding the product of colony displacement, yeast diameter (5 μm), and inverse time interval.

### *E*. *coli* sucrose concentration gradient

We decreased the motility of flagellated *E*. *coli* (O157:H7) by increasing the concentration of sucrose in the LB growth media to determine the μPAD assay’s sensitivity to displacement and growth rate measurement of a motile organism. We prepared 200 mL LB agar (20 g/L LB in DH2O; .1% ampicillin by volume) and segregated the sample into 9 parts to prepare the various sucrose concentrations (0, 100, 200, 300, 400, 500, 600, 700, and 800 mM) for the μPAD assay. We compared the μPAD assay against a “traditional” growth plate assay in which we prepared Petri dishes with the appropriate concentrations of sucrose in LB agar per the protocol suggested by Li and colleagues [16]. Given the diameter of *E*. *coli* is about 1 micron, we found the product of colony displacement, bacterium diameter, and inverse time interval to determine the growth rate of the colony in the given channel [28, 29].

### Microbial growth analysis

Regardless of the experiment run, we analyzed the microbial displacement within each of the same channels via the same methodology. We uploaded each picture to Adobe Photoshop CC 2018 and cropped the image around the interior border of the hydrophilic channel set. Since each channel set had an interior length of 10 cm, we changed the dimensions of the canvas such that the canvas length and image resolution corresponded to 10 cm and 242.6 pixels/cm, respectively. Then, we took advantage of the distinct edges of the microbial colonies against the agar surface by using the “Quick Selection Tool” feature to select the colony within each channel. We recorded the length of the microbial selection in pixels by using the “Properties” feature located on the right-hand pane of the Photoshop user interface. Finally, we converted this measurement into SI units with the resolution ratio established at the beginning of the process. We compiled the final recordings of microbial length to establish displacement and growth rate over the interval appropriate for their species.

## Results

### μPAD optimization

We optimized the linear channels of the μPAD for channel width, agar volume, and inoculation technique. We determined a priori that optimal conditions would be conducive to (1) consistent, flat growth fronts (i.e. Score 3), (2) sufficient, measurable microbial growth, and (3) easy agar inoculation. We assigned a Growth Score to each of the channels of the system to provide a semi-quantitative approach to assessing the system as a whole. Scoring is a common practice in immunohistology for group comparisons in research analysis [30]. In this experiment, we quantified the ways yeast grew within each channel by taking pictures of the growth fronts five days after yeast inoculation and noting shape differences according to the criteria described in Fig 3.

**Fig 5:**
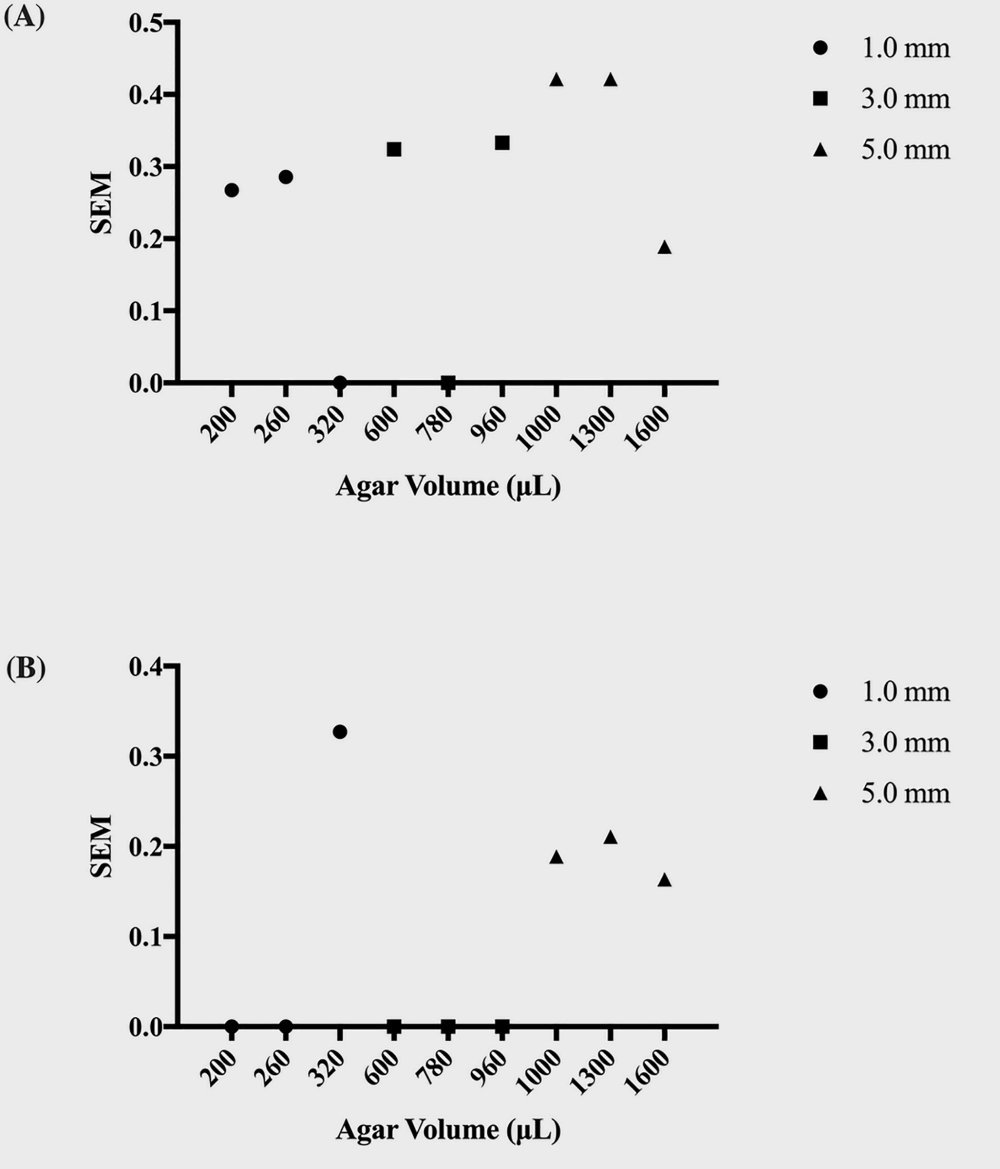
Growth score *SEM* for pipet and razor-blade microbial inoculation methods. The graphs show the standard error of measurement (*SEM*) for the growth scores of colonies inoculated via (A) pipet-inoculation and (B) razor blade-inoculation across different agar volumes. Note the improved consistency (*SEM* = 0) in growth score for the channels inoculated via the latter method.

Primarily, we considered any channel parameters that were not conducive to 100% flat microbial growth fronts to be unsuitable for the μPAD assay. By simple inspection, the razor-blade inoculation method vastly improved the growth scores and growth score standard error of measurement in nearly every channel over the pipet inoculation method (Figs 4 and 5). Thus, we continued with the razor-blade inoculation method for the remainder of the experiments. Regardless of inoculation technique, some agar and channel width combinations were more likely to exhibit spillage upon agar inoculation than others.

Per our criterion for consistent, flat growth fronts, we considered the channels filled with 200, 260, 600, 780, 960 μL agar as contenders for the final μPAD assay. However, we discarded 200 μL agar channels because the agar consistently dried out before the five-day interval of the trials. This may have had a diminished effect on yeast growth under these parameters. We conducted a one-way *ANOVA* to determine whether these different agar volumes (260, 600, 780, 960 μL) yielded a statistically significant difference on the mean displacement of yeast growth among these over the five days. The results suggested there was a nonsignificant relationship between the agar concentrations and mean microbial displacement, *F*(3, 6) = 2.971, *p* = .1188. Therefore, the remaining four channel conditions promoted sufficient, measurable microbial growth.

Finally, we considered ease of agar inoculation. We discarded any agar volumes that yielded a growth score of 0 in at least 25% of the trials. Since Score 0 indicates agar overflow in the channels (Fig 3A), we considered these agar volumes too difficult to consistently inoculate into the channels. Thus, we were left to consider channels with 780 and 960 μL agar, both of which were inoculated into channels with widths of 3 mm. Since we inoculated both volumes into channels of the same width, we decided to continue the protocol by inoculating the channels with 780 μL. We found smaller agar volumes were easier to inoculate in a shorter amount of time than larger volumes in the same channel width. Thus, we developed a μPAD assay implementing razor-blade inoculation of microbes onto 3 mm-wide channels with 780 μL agar.

### *S*. *cerevisiae* cycloheximide concentration gradient

**Fig 6:**
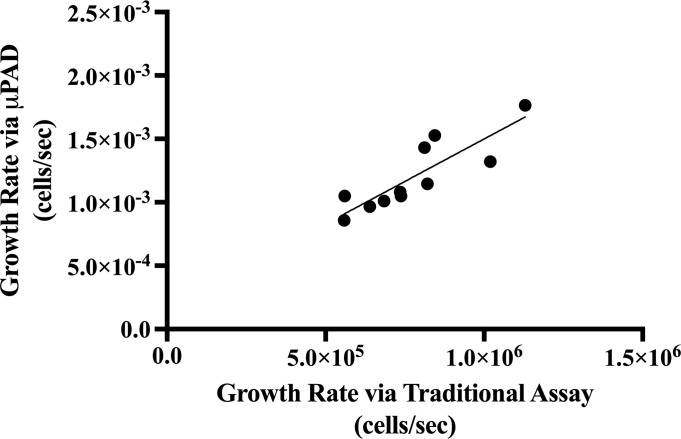
Comparison of growth rate measurements via traditional and μPAD assays for *S*. *cerevisiae* cycloheximide gradient. The relationship between the *S*. *cerevisiae* growth rate (cells/sec) measured by the traditional spectrophotometric method (x-axis) and the proposed μPAD method (y-axis) can be modelled by the equation, y = (1.34×10^(-9)) x + (1.59×10^(-4)). This model accounts for 74.35% of the variability in the data per *R*^*2*^ interpretations.

Through the *S*. *cerevisiae* cycloheximide gradient, we aimed to explore the sensitivity of the proposed μPAD assay to growth rate as compared to that of the traditional spectrophotometry assay. Fig 6 compares the trends in growth rate provided by the traditional assay against the those provided by the μPAD assay. The linear regression for the data indicates a correlation coefficient of *R* = 0.8623, suggesting a strong, positive correlation between the growth rates determined by the traditional and μPAD assays. Moreover, an *F*-test of overall significance revealed a significantly nonzero slope to the model relating the growth rates of the traditional and μPAD assays, *F*(1,9) = 26.09, *p* = .0006. We can reject the null hypothesis stating that the intercepts-only model is as good of a predictor of growth rate measured by the μPAD assay as the growth rate determined by the traditional assay. Thus, the μPAD assay is accurate enough to determine fluctuations in growth rate.

We compared the reliability of the traditional and μPAD assays by conducting an independent *t*-test of the relative errors of both assays. There was a nonsignificant difference between the mean relative standard errors of the traditional and μPAD assays (*p* = .7962). This suggests that the traditional spectrophotometry method was comparably variable in its measurement of yeast growth rate as the proposed μPAD method.

### *E*. *coli* sucrose concentration gradient

**Fig 7:**
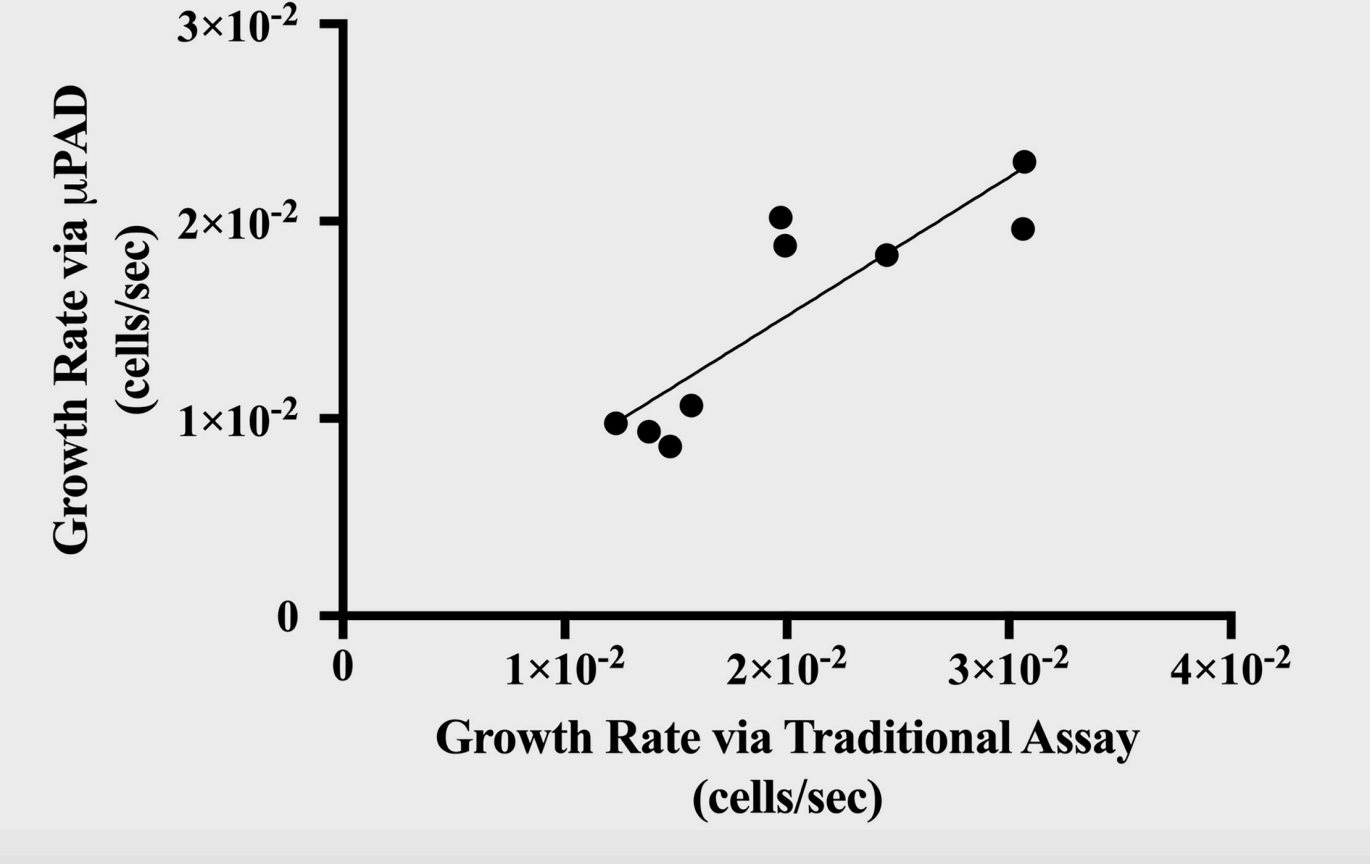
Comparison of growth rate measurements via traditional and μPAD assays for *E*. *coli* sucrose gradient. The relationship between the *E*. *coli* growth rate (cells/sec) measured by the traditional Petri dish method (x-axis) and the proposed μPAD method (y-axis) can be modelled by the equation, y = .7027x + .0011. This model accounts for 75.56% of the variability in the data per *R*^*2*^ interpretations.

We had similar goals for the *E*. *coli* sucrose gradient as the *S*. *cerevisiae* cycloheximide gradient. While both compare the sensitivity of the proposed μPAD assay with that of an accepted assay for growth rate, the *E*. *coli* sucrose gradient experiment applies the μPAD assay to a motile flagellar microorganism. Fig 7 compares the trends in growth rate provided by the traditional assay against those provided by the μPAD assay. The linear regression for the data indicates a correlation coefficient of *R* = 0.8692, suggesting a strong, positive correlation between the growth rates determined by the traditional and μPAD assays. Moreover, an *F*-test of overall significance revealed a significantly nonzero slope to the model relating the growth rates of the traditional and μPAD assays, *F*(1,7) = 21.64, *p* = .0023. We can reject the null hypothesis stating that the intercepts-only model is as good of a predictor of growth rate measured by the μPAD assay as the growth rate determined by the traditional assay. Again, the results support the assertion that the μPAD assay is accurate enough to determine fluctuations in growth rate, including that of motile microorganisms.

We compared the reliability of the traditional and μPAD assays by conducting an independent t-test of the relative errors of both assays. There was a nonsignificant difference between the mean relative standard errors of the traditional and μPAD assays (*p* = .0763). Similar to the yeast cycloheximide gradient, these results suggest that the traditional spectrophotometry method was comparably variable in its measurement of motile bacteria growth rate as the proposed μPAD method.

### Inter-device reliability

**Fig 8:**
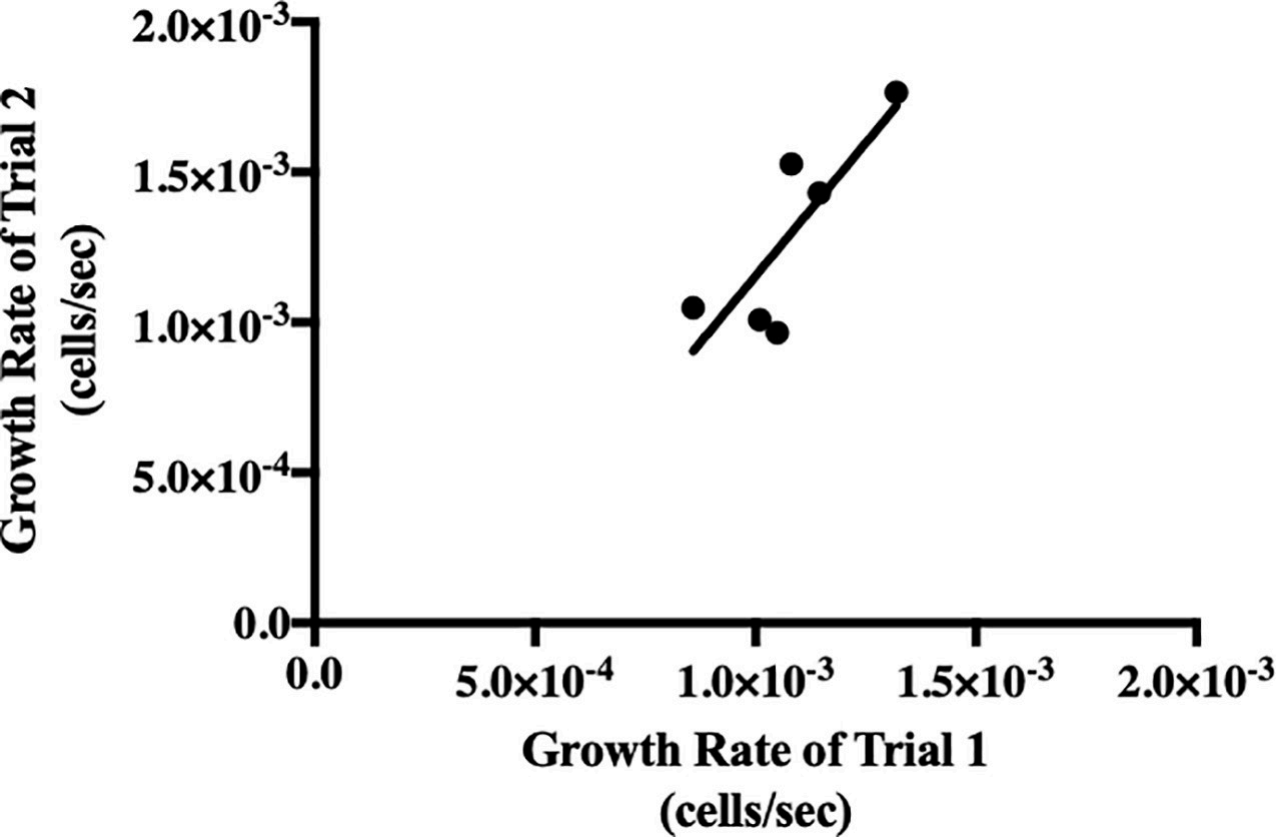
Comparison of growth rate measurements via two independent trials of *S*. *cerevisiae* cycloheximide gradients on the μPAD assay. The relationship between the growth rate (cells/sec) measured during Trial 1 (x-axis) and Trial 2 (y-axis) can be modelled by the equation, y = 1.77x + .0006151. This model accounts for 67.22% of the variability in the data per *R*^*2*^ interpretations.

To attest to the inter-device reliability, we conducted an independent t-test of the relative errors of two independently performed trials of the *S*. *cerevisiae* cycloheximide concentration gradient. These trials were conducted on different days with independently assembled μPAD devices. There was a nonsignificant difference between the mean relative standard errors of the two trials (*p* = .5535). Fig 8 compares the trends in growth rate provided by the two independently performed trials on the μPAD assays. The linear regression for the data indicates a correlation coefficient of *R* = 0.8199, suggesting a strong, positive correlation between the growth rates determined by the traditional and μPAD assays. Moreover, an *F*-test of overall significance revealed a significantly nonzero slope to the model relating the growth rates of the two trials, *F*(1,4) = 8.202, *p* = .0457. We can reject the null hypothesis, which supports our assertion that the μPAD assay is a reliable means of recording growth rate of microbial species.

## Discussion

In a field with growing demand for cost-efficiency and high-throughput methodology, microbiologists may find it difficult to integrate these aspects for growth assays with the means of traditional spectrophotometer/ microtiter plate and Petri dish techniques. The proposed μPAD method provides researchers, lab technicians, and students with the simple tools to develop an environmentally-friendly and versatile design for such needs. The experiments we conducted optimized the ideal parameters for linear growth assays and demonstrated the comparability of the traditional and μPAD assays for measuring growth rate for nonmotile and motile organisms.

Perhaps the most advantageous characteristics of the linear channel μPAD assays are their high-throughput designs and low-cost fabrication that produce relatively comparable growth displacement results to those achieved via traditional Petri dish and spectrophotometer assays. A cost analysis comparison of the traditional growth assays and μPAD assay highlights the benefit of the latter over the former. Specifically, the traditional method proposed and implemented here by Li and colleagues [26] for *E*. *coli* sucrose gradient experiments called for individual Petri dishes for each colony, which summed to 24 plates for triplet data sets. The μPAD method allows for quadruplet data sets to be collected in only four pipette boxes. Moreover, the compact design of the μPAD channels requires approximately 3% of the growth media required for the triplet data sets of the Petri dish assay. Therefore, a researcher could attain more data points with fewer materials via the μPAD assay.

Further, the device is suitable for facilities that have limited resources. The methods proposed in this study utilize materials that can also serve as common office supplies, specifically the paper and the printer. This is not unique to our design alone. In fact, Zhu and colleagues [31] developed a 2-D colorimetric μPAD assay via wax printing on chromatography paper to detect glucose, and Malekghasemi and colleagues [32] developed a 2-D calorimetric μPAD assay via inkjet printing on filter paper to detect urease. Similar to us, these parties took advantage of common office and laboratory supplies to produce μPADs. Once developed, the devices can withstand high temperatures that may be present in facilities with limited resources. The wax ink begins to melt/diffuse at 100°C. Given that this temperature abundantly exceeds that reached by any global climate, the printed channels will keep their integrity regardless of their facility of use. We recommend that the fully-constructed devices be handled in dry, sterile conditions after the autoclaving step to prevent contamination [33]. This substantiates the adaptability of this technique and design to labs of all financial backgrounds.

Of course, the cost-effectiveness and high-throughput aspects of our proposed μPAD assay would bear no weight if the protocol we developed were not effective. As the data suggests, the μPAD protocol we optimized, which concluded in an interior channel width of 3 mm, an agar volume of 780 μL/channel, and the razor-blade microbe inoculation method, yields growth rate results comparable to those achieved by traditional growth rate assays for nonmotile and motile microorganisms. The strong, positive correlations between the growth rates determined by the traditional method and μPAD method established by the *E*. *coli* sucrose gradient and *S*. *cerevisiae* cycloheximide gradient suggest the μPAD assay is accurate in its delineation of microbial growth rate. Moreover, the nonsignificant differences in standard error for the nonmotile and motile assay comparisons suggests there is comparable variability between the traditional and μPAD assays. Therefore, we conclude the proposed μPAD assay with linear channels to restrict microbial growth offers an accurate and reliable means for determining relative growth rate.

Growth assays are an integral part of microbiology and immunology experimental designs; thus, simple, yet effective, means of recording data are of top importance to researchers. Contemporary techniques rely upon expensive UV-Vis spectroscopy equipment. These spectroscopy methods exploit computer systems that automatically take data points in any indicated series of time intervals, be it every day or every few seconds, until the trial ends. This is a shortcoming of manual μPADs, which require a person to sterilize a work bench for appropriate pictures each time a data point is desired. However, the manual recording of data does offer the proposed μPAD method an advantage, nevertheless. Since we analyzed each channel image with Adobe Photoshop, we were able to note the smallest changes in gross colonial size down to the pixel. With each image sized to the area of the channels and customized to a resolution of 243 pixels/cm, the process allows for detection of changes in growth as small as .041 mm. This conferred us great precision in determining microbial displacement and, by extension, microbial growth rate. Furthermore, manual data recording is not unique to the proposed μPAD method. In fact, traditional growth assays that implement Petri dish methodology, as used in the *E*. *coli* sucrose gradient experiment, also require manual pictures and measurements. Sager and colleagues [34] justified use of videography to track microbial growth over time, the product of which could be digitally analyzed for data collection. Of course, these analyses require more profound understanding of computer software programming to properly construct an algorithm to delineate growth rate, which obstructs the simple design of our μPAD protocol.

The ultimate attraction of the proposed μPAD assay is its sheer versatility across a plethora of growth assay designs. A comparison of techniques for developing μPAD designs noted how wax printing is a rapid technique that takes advantage of easily accessible, commercially available equipment to print hydrophobic regions without exposing the channels’ hydrophilic regions to polymers or solvents [35, 36]. The only limitation of the method is the consideration of wax spreading that results from the melting of the printed design during μPAD preparation. After melting, the smallest channel width was measured at 561 ± 45 μm [35]. While this may be worthy of concern for electrochemical assays, this is hardly an issue for the proposed microbiological assay, where the optimal channel width was determined to be 3 mm for substantial colonial growth. With these parameters in consideration, the μPAD method can be molded to fit an assay. Since the agar fills the shape of the hydrophilic channels per capillary flow, the inoculum will grow to the shape of the printed μPAD design. Therefore, growth assays could implement branching, where a single colony inoculated at the center of the assay is forced to branch into different channels, each of which presents a different growth media environment. Such a design, which can be produced via any computer software with basic graphic design capabilities (Microsoft Office Word, Adobe InDesign, etc.), gives a holistic observation of gene surfing across the Fisher waves of each branch, a phenomenon explained by Hallatschek and Nelson [25].

Other assays could implement the exact linear μPAD design to investigate the behavioral changes in different colonial strain coexisting in a common environment (i.e. the channel) of a given microbial species. The simple μPAD assay supports preliminary analyses in the macroscale interactions between the different organisms with a technique far more cost-efficient than the Biomek FXp Laboratory Automation Workstation. Traditional fluorescence markers and dyes, such as GFP, bromocresol purple, and tetrazolium dyes, could facilitate the analysis of different strains of microbes during such a competition assay [37]. In essence, the proposed μPAD assay has the potential to be integrated into any microbiology experimental design where growth displacements, rates, and patterns on solid surfaces is the area of interest.

The proposed μPAD method allows researchers to develop personalized designs for growth assay on solid surfaces with incredibly low costs. The economic feasibility of wax printing in the lab or classroom setting promotes a virtually limitless supply of replicates and trials, which allow for error in design and procedural technique with little financial repercussion. Ultimately, μPADs merit consideration for replacement or integration into microbial growth assays that currently rely upon costly spectroscopy methodology.
